# AI based comparison of prefrontal cortex activity between piano-majors and non-piano-major musicians during score-based playing and Motif-improvisation

**DOI:** 10.1371/journal.pone.0337458

**Published:** 2025-12-26

**Authors:** Hyo Jee Kang, Su-cheon Lee, Yoobin Hwang, Jisun Ham, Manbok Park, Kang-moon Park

**Affiliations:** 1 Department of Music, Media Contents, and Biomedical Engineering, Korea National University of Transportation, Chungju, Republic of Korea; 2 Department of Electronics Engineering, Korea National University of Transportation, Chungju, Republic of Korea; 3 Department of Clinical and Counseling Psychology, Seoul, Republic of Korea; 4 Creative Minds Lab MAG, Raon Private Edu, Seogwipo-si, Republic of Korea; Air University, PAKISTAN

## Abstract

This study aimed to investigate how neural activation patterns differ between pianists and non-pianist musicians during piano performance tasks, using an LSTM-Autoencoder model applied to fNIRS data. A total of 22 participants, comprising both piano-majors and non-piano-majors, were involved in this study. Each participant’s performance data, collected during Score-based playing and improvisation tasks, was analyzed using a Long Short-Term Memory (LSTM) Autoencoder model. Reconstruction errors in specific brain channels, measured through Functional Near-Infrared Spectroscopy (fNIRS), showed group-level patterns—particularly in channels 1 and 15 (app. BA 45L, BA 45R)—but these differences did not reach statistical significance in inferential tests. These findings are therefore reported as exploratory and suggest distinct neural engagement patterns associated with varying levels of musical expertise.

## Introduction

Musical improvisation is widely regarded as one of the most highly creative forms of musical performance, involving the spontaneous generation of novel musical sequences in real-time [1,2]. Prior neuroimaging work indicates large-scale involvement of prefrontal, premotor, and temporal cortices and, in many cases, relative downregulation of dorsolateral prefrontal cortex (DLPFC) alongside increased medial prefrontal engagement during improvisation—patterns interpreted as reduced top-down monitoring that facilitate creative flow [3–7]. However, most prior studies have relied on block-averaged contrasts or general linear modeling, analytical choices that may obscure rapid, transient neural dynamics characteristic of live performance, particularly in highly trained musicians [3,8].

Expert piano-majors—those who have received formal instruction in secondary education or earlier and continuously practiced their instrument—show pronounced neuroplastic changes in auditory–motor integration and cortical representation, as demonstrated by Bangert & Altenmüller [9] and Bangert et al. [10]. However, the majority of existing research has focused on broad contrasts between musicians and non-musicians or employed block-averaged analyses that obscure rapid, expertise-specific neural fluctuations [11,12]. Critically, no study has yet compared the fine-grained, time-resolved neural dynamics of expert piano-majors versus non-piano-major musicians during motif-based improvisation, a form of improvisation centered on the spontaneous elaboration of short, thematically significant musical fragments.

Functional near-infrared spectroscopy (fNIRS) offers a practical alternative, allowing continuous monitoring of cortical hemodynamics during naturalistic piano performance Nonetheless, its application to musical creativity remains limited and mostly exploratory, as previous fNIRS studies predominantly rely on traditional statistical methods that average signals over extended intervals, thus failing to capture rapid temporal dynamics critical for understanding live creative processes [13–16]. While prior studies have provided valuable insights into musical creativity using block-averaged statistical methods, these approaches are inherently limited in capturing rapid, transient neural dynamics that characterize live performance. Although deep learning techniques have recently been applied to EEG and fMRI data to explore neural patterns, including aspects of musical creativity [17,18] such methods have not yet been applied to fNIRS recordings during real-time musical improvisation. This gap is significant because fNIRS allows for naturalistic, continuous monitoring of cortical hemodynamics, making it uniquely suited for capturing fine-grained temporal patterns that traditional statistical analyses cannot resolve. By leveraging a sliding-window LSTM-AutoEncoder, our study addresses this critical limitation, enabling autonomous detection of subtle, time-resolved neural dynamics and highlighting the novelty and necessity of an AI-driven time-series approach for understanding musical creativity.

To address this gap, we introduce an unsupervised sliding-window LSTM-AutoEncoder applied to high-resolution fNIRS time-series data recorded from 15 channels of the NIRSIT Lite system. Using reconstruction errors as an index of neural novelty, this approach autonomously detects subtle, transient patterns without requiring labeled data, allowing sensitive identification of expertise- and task-dependent dynamics.

The objective of this study is to investigate neural dynamics in expert piano-majors, building on the foundational work of Kang [19]. Specifically, we apply an unsupervised LSTM-AutoEncoder model for time-series neural data analysis to compare motif-improvisation with matched score-based playing, to identify subtle neural deviations overlooked by conventional block-averaged methods. This approach advances both the neuroscientific understanding of musical creativity and the development of neuro-inspired AI models emulating human creative cognition.

## Literature review

Research in neuroscience has particularly illuminated how various regions of the brain contribute to this complex task. Among these, the prefrontal cortex (PFC) has consistently been identified as a central hub, because it integrates executive control, working memory, and self-monitoring—functions that are indispensable for creative musical performance. Highlighting this pivotal role provides the rationale for beginning our literature review with the PFC. In particular, previous studies have indicated the importance of PFC in creative playing [4–6,19–22], and therefore this study will primarily explore the PFC regions.

### Dorsolateral Prefrontal Cortex (DLPFC)

The DLPFC is central to executive functions, including working memory, decision-making, and cognitive control. Research by Limb and Braun [4], and Jo and Kang [22] showed a decrease in DLPFC activity during improvisation, associated with reduced self-monitoring and enhanced creative flow. This decrease facilitates a state of cognitive flexibility, allowing musicians to generate spontaneous musical ideas without the constraints of over-monitoring their performance. The transient hypofrontality theory proposed by Dietrich and Kanso [23] further supports this by suggesting that creative states involve a temporary downregulation of prefrontal cortex activity. Ferreri et al. [24] found a decrease in DLPFC while listening to music compared to silence.

### Ventrolateral Prefrontal Cortex (VLPFC)

The VLPFC is implicated in processing complex, novel information and is crucial for adaptive behaviors. Located in the frontal lobes of the brain, primarily involved in cognitive functions like processing language and managing speech production. The VLPFC contributes broadly to language comprehension and is involved in higher-order cognitive processes, such as reasoning, planning, and problem-solving. Previous studies have shown that the ventrolateral prefrontal cortex (VLPFC) plays a critical role in integrating diverse information during creative tasks such as musical improvisation. This region is particularly active when musicians must suppress habitual responses and generate novel solutions, which are essential for high-level improvisation [5,7].

### Inferior Frontal Gyrus (IFG) and Broca’s Area

The Inferior Frontal Gyrus (IFG) is part of the VLPFC and includes areas such as Broca’s area, specifically involved in language production and processing, illustrating the specific roles these areas play within the broader prefrontal cortex functionalities [23]. Within the IFG, Broca’s area, predominantly located in the left hemisphere and including Brodmann areas 44 and 45, is critically involved in language production, underlining the region’s importance in speech articulation and the complex motor functions required for producing speech. The various studies referenced in the provided citations confirm and expand upon this idea by highlighting the involvement of Broca’s area (specifically the inferior frontal gyrus) in both language processing and musical creativity [25–27]. During improvised rock guitar performances, Tachibana [5] demonstrated increased activity in Broca’s area and its right hemisphere homologue. This activity suggests that Broca’s area is involved in managing complex, non-verbal auditory processing during musical improvisation, similar to how it functions in language syntax. This highlights the overlap between language and music in cognitive processing, where both domains require the integration of complex, structured sequences.

## Methodology

### Participants

As shown as Table 1, participants in this study were a group of 22 classically trained musicians, officially majoring in music institutes such as music colleges or conservatoires from higher than high school level. They were recruited through targeted announcements on music college websites and societies focused on piano pedagogy. This recruitment strategy ensured that all participants possessed a strong foundation in classical music, allowing for an in-depth analysis of neurophysiological aspects among musicians with varying skill levels. The cohort consisted of 4 males and 18 females, with ages ranging from 19 to 58 years (M = 35.5, SD = 9.99). Participants’ years of piano training varied from 1 to 50 years (M = 16.51, SD = 10.23). Sixteen participants were piano-majors, while the remaining six were non-piano majors. As shown as Table 2, for piano-majors (n =16), the mean age was 35.69 years (SD = 8.87), with piano training ranging from 10 to 50 years (M = 21.4, SD = 10.81). As shown as Table 3, non-piano-major musicians (n =6) had an average age of 35 years (SD = 14.25), with piano training ranging from 10 to 50 years (M = 5.33, SD = 3.50). In the study group of non-piano-major musicians (non-piano-majors), there are 4 females and 2 males. The ages range from 19 to 51 years, with an average age of 35 years and a standard deviation of approximately 14.25. The years of piano training range from 1 to 10, with an average of approximately 5.33 years and a standard deviation of about 3.50.

**Table 1:** Participant demographics and musical background.

Description	Total	Males	Females	Age (mean ± SD)	Age (range)	Piano Years (M±SD)	Piano Years (range)
Participant Distribution	22	4	18	35.5 ± 9.99	19–58	16.51 ± 10.23	1–50

**Table 2:** Statistics of Piano major participants.

Description	Total	Males	Females	Age				Piano years			
				Mean	SD	Min	Max	Mean	SD	Min	Max
Participant distribution	16	3	13	35.69	8.87	25	58	21.4	10.81	10	50

**Table 3:** Statistics of non-Piano major participants.

Description	Total	Males	Females	Age				Piano years			
				Mean	SD	Min	Max	Mean	SD	Min	Max
Participant distribution	6	2	4	35	14.25	19	51	5.33	3.50	1	10

### Experimental design

A 60-minute workshop was conducted by a professional improvisation instructor, who led all participants through exercises focusing on arpeggio-based improvisation, thematic improvisation, and emotional expression. Previous research indicates that shorter sessions may not provide enough time for classical musicians, often less experienced with improvisation—to overcome anxiety and build creative confidence [28]. The workshop aimed to familiarize participants with improvisation techniques and reduce performance anxiety. We tested score-based playing, motif improvisation, and free improvisation in random order. In this study, we performed a comparative analysis only between score-based playing and motif improvisation, while previous studies have demonstrated significant neural differences between score-based playing and free improvisation [19,22]. Table 4 summarizes the timing of task and rest periods. Rest periods were inserted between experimental tasks to allow hemodynamic activity to return to baseline, ensuring a clear contrast between task-induced activations and the neutral state [24,29].

**Table 4:** Period of one cycle (score-based playing, Motif-improvisation, free-improvisation) of experiment.

Activity	Rest	Preparation	Rest	Play	Rest	Total time
Time (duration)	30 s	30 s	10 s	60 s	30 s	160 s (2 min 40 s)

#### Score-based playing.

Score-based playing refers to performance practice based on the faithful realization of notated musical scores. We allowed participants to practice without memorization for 3 days and experiment while playing with the score.These functions as a baseline to compare with motif-improvisation [30,31]. We selected Muzio Clementi’s Sonatina in C Major for both piano-majors and non-piano-majors, considering its accessible classical sonata form and 4/4-time signature. The piece was suitable for mastery within a three-day preparation period, allowing participants to familiarize themselves with the music and focus on performance without the cognitive load of memorization. This preparation time struck a balance between familiarity and tension, avoiding sight-reading, which could introduce anxiety [32], or memorization, which could lead to over-familiarity and hinder improvisational focus [33].

#### Motif-improvisation.

Motif refers to a short musical fragment or sequence that possesses thematic significance and symbolic meaning within a compositional context. It functions as a foundational unit that encapsulates the identity of a musical work. Motif-Improvisation, therefore, denotes a performance practice in which a single motif is initially presented and subsequently developed through spontaneous, creative elaboration in real time [34].

Motif-based improvisation was designed to provide a semi-structured creative task that bridges fully composed performance and free improvisation. This format avoids the confounds introduced by large variations in tempo, meter, and harmonic language inherent in jazz or free improvisation, which can significantly alter both motor execution and neural dynamics [16,35]. Although constrained, motif-based improvisation engages key cognitive processes such as auditory imagery, predictive timing, and hierarchical sequencing, which are known to activate prefrontal and language-related brain areas, including Broca’s area [36,37]. The first two bars of the Score-based playing piece serve as the motif material for improvisation. This design was inspired by classical thematic development practices and adapted to be more accessible for non-improvisers. Using C Major and a right-hand–focused motif reduces technical complexity, thereby allowing performers to focus more on creative transformation and expressive intent rather than motor accuracy. This approach aligns with findings that constrained improvisation can still evoke complex neural and creative processes, especially in classically trained musicians unfamiliar with improvisation idioms [38].

**Fig 1:**
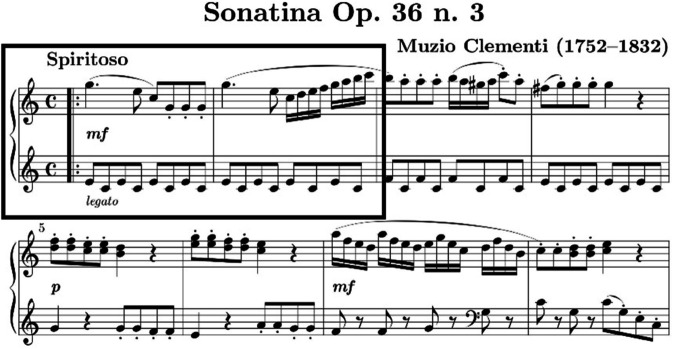
Beginning of Clementi Sonatina Op. 36, No. 3 (1st movement).

As shown in Fig 1, for Score-based playing, participants play through for 60 seconds. For Motif-Improvisation, participants play the first two bars and keep playing improvisationally for 60 seconds.

### fNIRS data

**Fig 2:**
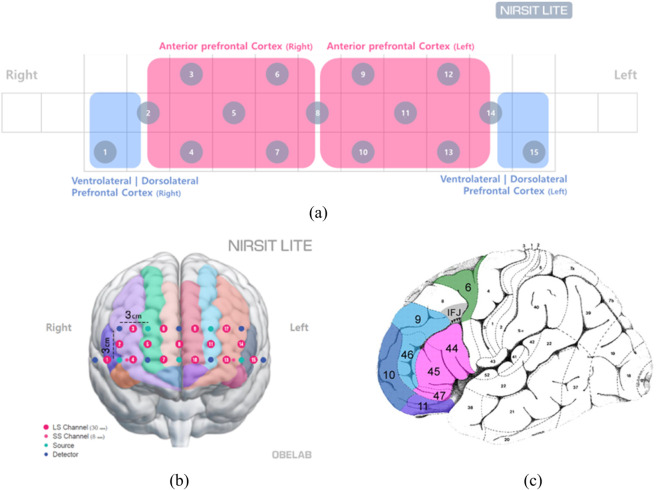
NIRSIT Lite channels and cortical regions. (a) NIRSIT Lite channels [18], (b) frontal brain surface regions [18], and (c) Brodmann areas at the prefrontal cortex [21].

Fig 2 (a) represents the functional division of PFC where the channel of NIRSIT Lite measures. (b) is approximately on the shown brain surface [39]. Measurements will be taken from approximately 15 channels from Brodamann Area (BA) 10, 46, 45. (c) shows Brodamann’s area from lateral view of the brain including the orbitofrontal cortex (BA 11, purple), ventrolateral prefrontal cortex (VLPFC, BA 44/45/47, pink), inferior frontal junction (IFJ, gray), dorsolateral prefrontal cortex (DLPFC, BA 9/46, light blue), anterior prefrontal cortex (BA 10, dark blue), and dorsal premotor cortex (BA 6, dark green) [28,40]. fNIRS and MIDI recordings were conducted using the NIRSIT LITE of OBELAB, Inc and Logic Pro. During the fNIRS scans, the participants played a YAMAHA P125 keyboard with 88 keys. MIDI data was collected simultaneously. During scanning, the participants’ performance on the keyboard was recorded with their consent. Auditory feedback from the piano was provided to the participants through speakers. They were instructed not to move their heads during the playing. During all three conditions, participants were instructed to direct their gaze forward. We collected neural data using the fNIRS system, focusing on the PFC, known for its involvement in cognitive flexibility, decision-making, and creative processes [28]. Functional near-infrared spectroscopy (fNIRS) is a non-invasive technique ideal for studying PFC activity in musicians, as it effectively captures hemodynamic responses and allows for brain activity measurement in naturalistic settings, thus enhancing ecological validity [24,41]. Its sensitivity to cognitive control and creativity makes it particularly useful for investigating the neural mechanisms underlying musical improvisation and performance [24,42,43]. Data were recorded at a sampling rate of 8 Hz (8 data points per second). Hemodynamic responses were assessed using changes in oxyhemoglobin (HbO) and deoxyhemoglobin (HbR) concentrations, filtered in real-time to remove motion artifacts. These physiological signals were synchronized with MIDI performance data, ensuring alignment between brain activity and musical execution. For real-time fNIRS data processing, we used OBELAB’s proprietary software, designed specifically for the NIRSIT LITE system. This software allowed for the efficient acquisition and processing of neural data, providing seamless integration with MIDI data to ensure precise synchronization between brain activity and musical performance [15,39,44–46].

### Data set

An LSTM-AutoEncoder model is used to facilitate this comparison, wherein the model is trained on the outcomes of Score-based playing, and the reconstruction error for Motif-Improvisation is subsequently measured. This methodology identifies channels exhibiting anomalies, with elevated reconstruction errors indicating the presence of variations in the data patterns. Due to the varying lengths of the collected data, all data samples were adjusted to a uniform length of 1800 time points(steps) for (the purpose of) training.

### Neural network model structure

**Fig 3:**
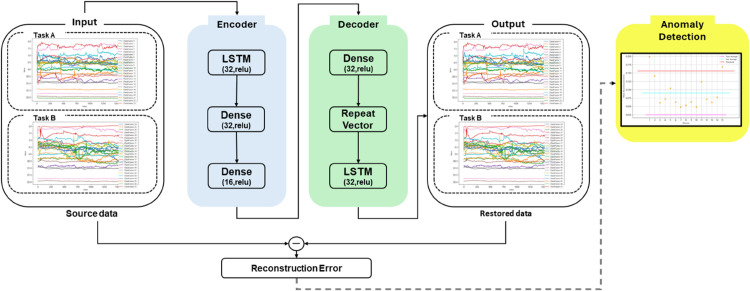
Architecture of the LSTM-AutoEncoder model.

Previous studies have demonstrated that LSTM-AutoEncoder models generally outperform standard AutoEncoder models when dealing with large volumes of temporal data, particularly in anomaly detection tasks [17,28,47]. This is due to LSTM’s ability to capture long-term dependencies in time series data [48]. In this study, we also leverage the advantages of the LSTM-AutoEncoder to implement a model suitable for the characteristics of time series data. Traditional statistical methods, such as mean signal comparison or GLM-based analysis, have been widely used in fNIRS studies to evaluate brain activation differences across conditions [49]. However, these approaches often rely on assumptions of stationarity and may fail to capture nonlinear temporal dependencies or subtle individual variability, especially in dynamic tasks like musical improvisation. In contrast, deep learning models such as LSTM-AutoEncoders can learn complex temporal patterns directly from data without requiring handcrafted features [50,51], making them particularly suitable for identifying latent deviations in neural activity. The architecture of the LSTM-AutoEncoder utilized in this research is illustrated in Fig 3. Considering the temporal nature of the collected data, the LSTM-AutoEncoder model was implemented by combining Long Short-Term Memory (LSTM), which is suitable for time series data learning, with the AutoEncoder, a model designed for unsupervised learning [23,25,52–54]. The AutoEncoder reduces the input data via the encoder section and reconstructs it using the decoder section. The discrepancy between the reconstructed data and the original data is called the reconstruction error. Leveraging these characteristics, AutoEncoder models are utilized in anomaly detection to identify patterns that differ from the training data [55–57]. In the context of neuroimaging, recent studies have demonstrated the utility of LSTM-based AutoEncoder frameworks in detecting abnormal temporal patterns in EEG and fNIRS signals, outperforming conventional methods in sensitivity and temporal resolution [58–60]. Our approach builds on these findings by applying an LSTM-AutoEncoder to identify neural deviations during improvisational tasks relative to baseline (score-playing) conditions. The AutoEncoder was first trained on Task A (Score-Playing) data to establish a normal baseline. Task B (Improvisation) data were then tested for deviations using reconstruction error analysis. In the encoder section of Fig 3, the input data is processed sequentially through LSTM layers, which consist of 64 and 32 units and utilize ReLU as the activation function. The data is then compressed into a latent space that reflects low-dimensional compressed data through Dense layers with 32 and 16 units. The data that has been compressed by the encoder is restored through the decoder. It is transformed into an appropriate sequence length for the decoder using the RepeatVector and subsequently processed through a Dense layer. Finally, it is reconstructed through the LSTM layer with 32 units, and the reconstruction error is assessed.

## Experimental results

This study utilized the Python programming language for data preprocessing and model implementation, while the TensorFlow framework was employed for training and optimizing deep learning models. Designed for efficient neural network computations, TensorFlow provides GPU acceleration and high scalability, which contributed to improving the efficiency of model training and evaluation during the experiments. This enabled the effective exploration of various neural network architectures and facilitated performance comparisons throughout the study

### Hyperparameters.

The hyperparameter values used in the learning model are shown in Table 5. In this study, mean square error (MSE) was used as the loss function. MSE is a metric that measures the mean squared difference between the predicted value and the actual value of the model, as defined in (1).

$$MSE=\frac{1}{n}\sum_{i=1}^{n}(y_{i}-{\hat{y}}_{i}{)}^{2}$$ (1)

**Table 5:** Final algorithm parameter settings. Summary of the hyperparameters used to train the LSTM-AutoEncoder model.

Name	Value
Loss function	MSE
Epochs (number)	200
Batch size (samples)	1024
Optimizer	Adam

Mean Squared Error (MSE) is particularly sensitive to large discrepancies, effectively emphasizing substantial differences between predicted and actual values. This characteristic is instrumental in enhancing overall predictive accuracy by minimizing significant errors within the model. Furthermore, MSE’s differentiable nature is advantageous for optimization algorithms, such as gradient descent, facilitating smoother convergence. Consequently, MSE is a suitable loss function for a variety of machine learning models, including those used in regression problems and neural network training. The experiments were conducted five times for each of the window sizes 15, 20, and 25, and the results were averaged and summarized in Table 6. Among these, the window size of 20 exhibited the lowest reconstruction errors, with the train and test reconstruction errors being approximately 0.03 and 0.09, respectively. Therefore, this window size was used in the subsequent analysis.

**Table 6:** Window sizes and corresponding train and test reconstruction errors.

Window size	Train reconstruction error	Test reconstruction error
15	0.04151	0.11382
**20**	**0.03002**	**0.09045**
25	0.03590	0.10850

### Training results.

**Fig 4:**
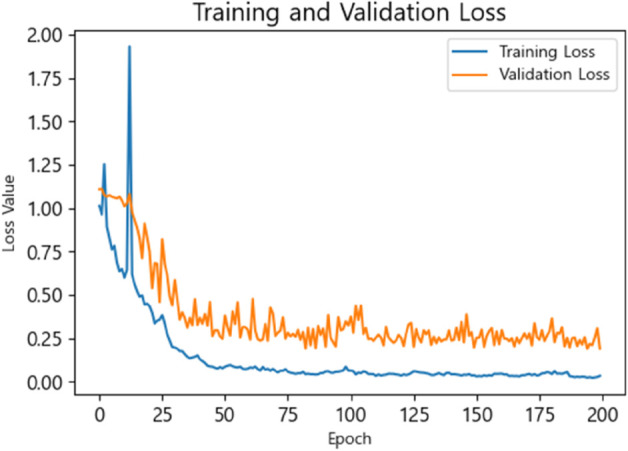
Training and validation loss.

As a result of training on Task A for all participants, it was observed that both the training loss and validation loss converged, as illustrated in Fig 4. As shown in Table 7, the relatively high training loss is approximately 0.039, while the validation loss is about 0.245. This may be attributed to the complexity of the data.

**Table 7:** Training and validation losses at a window size of 20.

Window size	Training loss	Validation loss
20	0.039	0.245

### Analysis results.

As shown in Table 8, when the window size was set to 20, reconstruction errors of approximately 0.25 and 0.18 were observed in channels 1 and 15, respectively.

**Table 8:** Analysis Result 1. Average Channel-wise reconstruction error for the entire dataset.

Window size	Channels with highest reconstruction errors
20	1, 15

As an additional analysis of Result 1, we examined the differences in channels between piano-majors and non-piano-majors. As shown in Table 9, with a window size of 20, significant reconstruction errors were observed in channels 1 and 15 for piano-majors. In contrast, for non-piano-majors, reconstruction errors of approximately 0.17 and 0.135 were observed in channels 2 and 5, respectively.

**Table 9:** Analysis Result 2. Average Channel-wise reconstruction error of data from pianists and non-pianists for Task A and B.

Window size	Channels with highest reconstruction errors	
	Pianists	Non-pianists
20	1, 15	None

## Discussion

### Comprehensive analysis

**Fig 5:**
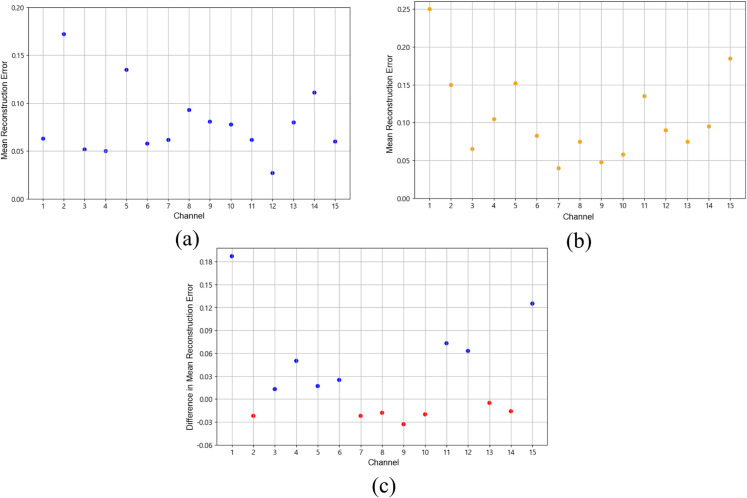
Channel-wise reconstruction errors. (a) Average for pianists, (b) average for non-pianists, (c) overall average across all participants.

In Fig 5(a), the LSTM-AutoEncoder model trained on Task A with a window size of 20 showed the largest discrepancies in channels 1 and 15 for pianists’ Task B. In contrast, Fig 5(b) displays the average reconstruction errors for non-pianists, showing relatively large discrepancies in channels 2 and 5. However, due to the insufficient data for non-pianists, the results were highly variable, making it difficult to draw definitive conclusions. Fig 5(c) provides a visual comparison of the average reconstruction errors across different channels for both pianists and non-pianists. As shown in the figure, channels 1 and 15 exhibited differences greater than 0.10. Nevertheless, inferential tests conducted on an *a priori* BA45 ROI (channels 1 and 15) with robust summaries (median log-MSE, trimmed mean, and 90th percentile) did not reach statistical significance (Welch’s *t*-test, Mann–Whitney *U*, and label-permutation; all *p* > 0.28), and effect sizes were small-to-moderate. On the other hand, the red dots indicate that the average reconstruction errors for non-pianists were larger, though the differences were not as pronounced. These descriptive patterns should therefore be interpreted as exploratory.

To formally assess the descriptive patterns in Fig 5, we (channels 1 and 15) used three robust summaries of the LSTM-AutoEncoder reconstruction error (median log-MSE, 10% trimmed mean log-MSE, and 90th-percentile log-MSE). None of the tests reached statistical significance at the group level (Welch’s *t*, Mann–Whitney *U*, and label-permutation with 10,000 iterations; all *p* > 0.28). For the most discriminative summary (90th-percentile log-MSE), we observed *p* = 0.283 (Welch’s *t*), *p* = 0.494 (Mann–Whitney), and permutation *p* = 0.297, with small-to-moderate effect sizes (Cohen’s *d* = 0.521; Cliff’s δ=0.208; pianists *n* = 16, non-pianists *n* = 6). These findings indicate that the visually apparent discrepancies—particularly at channels 1 and 15—should be interpreted as exploratory trends rather than conclusive group effects, likely reflecting limited power due to the small, imbalanced sample and inter-subject variability in fNIRS signals.

### Finding of neural mechanisms in improvisation

The elevated reconstruction errors observed in Channel 1 (BA45R) during improvisation (although not statistically significant in our inferential analyses) suggest a heightened neural engagement in tasks that require cognitive flexibility, motor inhibition, and action updating—functions strongly associated with the right VLPFC. This area’s involvement in improvisation highlights its role in managing the unpredictability and spontaneity inherent in creative tasks. Similarly, the findings in Channel 15 (BA45L) indicate that Broca’s area, traditionally known for its role in language production, is also critically engaged during musical improvisation. We note that these interpretations are exploratory given the non-significant statistical tests, but they suggest that the left IFG plays a pivotal role in the creative manipulation of musical elements, aligning with its known functions in syntactic processing and complex cognitive tasks. Interestingly, these findings support a growing body of research suggesting that improvisation, whether in music or language, involves complex and dynamic neural processes across both hemispheres of the brain, fostering the flexibility and adaptability essential for creative expression. Our findings align with those of Tachibana [5], who also observed significant activation in BA45L and BA45R during musical improvisation, particularly in improvisation as compared to more formulaic playing. The above study finds the averaged hemodynamic responses in the BA45 region during Improv and Formulaic task conditions.

The reason why piano-majors tended to show more consistent (though not statistically significant) neural response, especially in the context of creative musical tasks, can be attributed to their extensive training. Piano-majors undergo rigorous practice that fine-tunes their motor and cognitive skills, particularly those involving the integration of complex motor sequences and auditory feedback. This training likely enhances the connectivity and efficiency of neural networks associated with both musical and linguistic processing, making them more adept at engaging these regions during improvisation. This is in contrast to non-piano-majors, whose neural engagement in these regions was less consistent, likely due to their varied primary instruments, limited familiarity with the piano, and the small sample size. These factors introduce confounds that require further exploration, such as how differences in instrument familiarity influence the engagement of the prefrontal cortex in creative musical tasks. These factors introduce biases that make it challenging to detect stable neural responses during improvisation among non-piano-majors. The patterning in Channels 1 and 15, particularly in piano-majors, suggest a potential relationship between symmetrical brain structures and their connectivity. Since both Channel 1 and Channel 15 are located in the triangular part of the IFG, they likely share similar functional responsibilities, primarily related to language processing, both verbal and non-verbal, as well as cognitive control. Although they are located symmetrically on opposite hemispheres, their functional contributions could be asymmetrical. The left IFG (Channel 15) is more involved in language production and complex verbal tasks, while the right IFG (Channel 1) contributes to broader cognitive functions, including non-verbal communication and possibly emotional processing Studies have shown that high-level cognitive functions often require the coordination of both hemispheres [5,23]. The symmetrical activity might reflect the brain’s need to integrate various forms of information—such as timing, rhythm, and melody in the case of music—requiring efficient communication between the hemispheres. This is particularly true for tasks like improvisation, which involve both planned and spontaneous elements. This difference appears to be due to brain plasticity. Consistent practice has been shown to alter brain activity in both healthy individuals and patients with brain damage or neurological conditions [61–63]. More specifically, various studies have explored the effects of musical training on both structural and functional changes in the brain, highlighting its role in enhancing brain plasticity. These studies show that musical training increases connectivity between brain regions, improves linguistic and auditory abilities, and has positive effects on cognitive and neurophysiological functions [64–68]. Despite the unfamiliarity classical musicians may have with improvisational playing, training as a professional piano-major still helps bridge the gap between non-piano-major musicians during motif variation.

## Conclusions

This study explored neural activity patterns between motif improvisation and score performance using an AI-based autoencoding method. The error-based perspective may reveal patterns not readily captured by simple activation/deactivation analyses. However, at the group level we did not observe statistically significant differences under a priori ROI analyses with robust summaries and permutation testing; effect sizes were small to moderate. Given that music is a temporal art, it is natural for neural activity to fluctuate dynamically in response to musical features, and the error detection approach may provide a more nuanced analysis than a simple comparison of activation. Descriptively, pianists tended to show higher reconstruction errors in the right and left IFG triangularis regions (BA45R, BA45L), but these trends did not reach statistical significance in our dataset. In contrast, non-pianists showed greater variability, limiting interpretability. Accordingly, our findings are exploratory and should be interpreted with caution given the small and imbalanced sample and inter-subject variability in fNIRS signals. This work offers a complementary perspective on neural differences in creative musical tasks via error detection, and future research should validate these observations using within-subject designs, larger and balanced samples, and complementary baseline models.
